# Proteomes of native and non-native symbionts reveal responses underpinning host-symbiont specificity in the cnidarian–dinoflagellate symbiosis

**DOI:** 10.1093/ismejo/wrae122

**Published:** 2024-07-11

**Authors:** Amir Mashini, Clinton A Oakley, Lifeng Peng, Arthur R Grossman, Virginia M Weis, Simon K Davy

**Affiliations:** School of Biological Sciences, Victoria University of Wellington, Wellington 6140, New Zealand; School of Biological Sciences, Victoria University of Wellington, Wellington 6140, New Zealand; School of Biological Sciences, Victoria University of Wellington, Wellington 6140, New Zealand; Biosphere Sciences and Engineering, The Carnegie Institution for Science, Stanford, CA 94305, United States; Department of Integrative Biology, Oregon State University, Corvallis, OR 97331, United States; School of Biological Sciences, Victoria University of Wellington, Wellington 6140, New Zealand

**Keywords:** symbiosis, dinoflagellates, symbiosis establishment, specificity, proteomics, coral reefs, Symbiodiniaceae, Exaiptasia, Breviolum, Durusdinium

## Abstract

Cellular mechanisms responsible for the regulation of nutrient exchange, immune responses, and symbiont population growth in the cnidarian–dinoflagellate symbiosis are poorly resolved, particularly with respect to the dinoflagellate symbiont. Here, we characterized proteomic changes in the native symbiont *Breviolum minutum* during colonization of its host sea anemone *Exaiptasia diaphana* (“Aiptasia”). We also compared the proteome of this native symbiont in the established symbiotic state with that of a non-native symbiont, *Durusdinium trenchii*. The onset of symbiosis between Aiptasia and *Breviolum minutum* increased the accumulation of symbiont proteins associated with the acquisition of inorganic carbon and photosynthesis, nitrogen metabolism, micro- and macronutrient starvation, suppression of host immune responses, tolerance to low pH, and management of oxidative stress. Such responses are consistent with a functional, persistent symbiosis. In contrast, *D. trenchii* predominantly showed elevated levels of immunosuppressive proteins, consistent with the view that this symbiont is an opportunist that forms a less beneficial, less well-integrated symbiosis with this model anemone. By adding symbiont analysis to the already known responses of the host proteome, our results provide a more holistic view of cellular processes that determine host-symbiont specificity and how differences in symbiont partners (i.e. native versus non-native symbionts) may impact the fitness of the cnidarian–dinoflagellate symbiosis.

## Introduction

The success of coral reefs is dependent on their obligatory endosymbiotic relationship with photosynthetic dinoflagellate algae from the family Symbiodiniaceae [1]. The symbionts translocate photosynthetic products (e.g. glucose, amino acids, and fatty acids) to the host, and in return, the host provides the symbiont with inorganic carbon, nitrogen, phosphorus, various micronutrients, and shelter from an environment that can be hostile [2]. The family Symbiodiniaceae contains 11 described genera that are genetically, physiologically, and ecologically diverse [3–5]. Moreover, they exhibit different host specificities, with some forming symbioses with a much wider range of host species than others [6]. The mechanisms that underlie host-symbiont specificity have not been fully described; however, metabolic exchange and cellular recognition between the partner organisms are important determining factors [7, 8].

The cnidarian–dinoflagellate symbiosis is susceptible to changes in environmental conditions, especially conditions that elicit physiological stress. Upon exposure to thermal stress, the dinoflagellate symbionts may be lost from the host tissue, a process known as “coral bleaching” [9]. Some species of Symbiodiniaceae are more resistant to temperature anomalies, which can confer more thermal tolerance to the holobiont (i.e. the whole symbiosis) [10, 11]. *Durusdinium trenchii* is a host “generalist” that can persist through high temperatures and become a dominant symbiont after bleaching events, but may not allow for optimal fitness of the host under relatively “non-stressful” conditions; cnidarian hosts harbouring *D. trenchii* exhibit slower rates of growth and calcification, an increased immune response, and elevated levels of oxidative stress [12–14]. Symbiont identity is therefore crucial for determining the fate of the holobiont and, hence, the overall health of the reef ecosystem. Indeed, some corals acclimate to changing environmental conditions by processes described as “symbiont switching” (i.e. acquiring symbionts *de novo* from the environment) or “symbiont shuffling” (i.e. changing the relative abundance of the existing symbiont communities within colonies) [12]. Developing a detailed knowledge of processes involved in symbiosis establishment, specificity, and dysfunction is therefore important for predicting how coral reef ecosystems will be impacted by climate change, and may even suggest potential mitigating interventions [7]. The majority of studies on cnidarian–dinoflagellate associations have focused on the animal host rather than on the symbiont [15–19]. We must begin to fill this information gap if we are to understand the physiological and metabolic integration between the symbiont and its host, as well as the signalling events that coordinate this integration.

*Exaiptasia diaphana*, commonly known as “Aiptasia,” is a widely adopted model organism for dissecting cnidarian–dinoflagellate interactions crucial for a successful symbiosis [20]. Across the Indo-Pacific region, Aiptasia naturally harbours *Breviolum minutum*; however, the dominant algal symbionts in Florida are species of *Symbiodinium*, *Breviolum*, and *Cladocopium* [21]. Under experimental conditions, Aiptasia can establish a symbiosis with numerous different symbiont species. Although non-native symbionts may be less beneficial to the host [14, 22]. Currently, we have little knowledge of how the physiology of the symbiont responds to colonization of a non-native host, although there have been a limited number of transcriptomic, proteomic, metabolomic, and lipidomic studies that have identified differences in photosynthesis, transmembrane transport, cell division, and signalling processes between free-living and symbiotic states, little is still known about the events that define the early stages of host colonization [23–28]. Elucidating symbiont-specific responses that underlie symbiosis establishment and host-symbiont specificity will reveal the biological underpinnings of a successful symbiosis and the potential for corals to adapt to climate change by altering their algal partners.

We compared and contrasted the proteomes of the native *Breviolum minutum* and non-native *D. trenchii* over time following the colonization of Aiptasia. The proteome of *B. minutum* was characterized across multiple time-points during host colonization and compared to the proteome of *D. trenchii* in the fully-established symbiotic state. We hypothesized that native symbionts would show a faster and more pronounced increase in the abundance of proteins associated with symbiosis establishment, cell proliferation, metabolic exchange, and inter-partner signalling, and that these would indicate key proteins and mechanisms of a successful symbiosis. Conversely, we proposed that the non-native symbiont would exhibit a lag or lack of these proteomic shifts, reflecting its incomplete integration into host metabolism.

## Materials and methods

### Experimental organisms

Two Symbiodiniaceae species—the Aiptasia native *B. minutum* and the non-native *D. trenchii*—were grown for eight weeks prior to experimentation in Guillard’s *f*/2 medium in 0.22 μm-filtered seawater (FSW), at 25°C and 100 μmol photons m^−2^ s^−1^ irradiance on a 12 h:12 h light:dark cycle. *B. minutum* (culture identifier: FLAp2) was originally isolated from Aiptasia, and *D. trenchii* (culture identifier: Ap2) was isolated from an unknown anemone in Okinawa, Japan. The identity of these cultures was confirmed by genotyping methods as described in Mashini *et al.* [29]. These culture strains are available from the Cawthron Institute’s Culture Collection of Microalgae (Nelson, New Zealand) with the identifiers CAWD462 (*B. minutum*) and CAWD463 (*D. trenchii*).

Aiptasia specimens (*n* = ∼200) were taken from a clonal laboratory stock (strain identifier NZ1; originally from the Indo-Pacific region) and rendered aposymbiotic (i.e. symbiont-free) by incubating them in 0.19 mM menthol with 5 μM 3-(3,4-dichlorophenyl)-1,1-dimethylurea (DCMU), a specific inhibitor of photosynthetic electron transport (blocks electron flow out of Photosystem II), for 4 weeks [30]. The absence of symbionts was confirmed via a lack of chlorophyll autofluorescence, which was assessed by confocal microscopy (100× magnification, Olympus Provis AX70). All anemones were fed twice per week with *Artemia* sp. nauplii and maintained in the dark at 25°C for at least two months prior to using them for experiments. Animals used in this study can be obtained without restriction by contacting the authors.

### Inoculation of Aiptasia with symbiotic algae

Aposymbiotic anemones (*n* = ~400) were starved for one week prior to inoculation with algal cultures in 250-ml containers (20 anemones per container). FSW was refreshed prior to inoculation. One drop of symbiont cell suspension, concentrated by centrifugation to ~3 × 10^6^ cells ml^−1^ and mixed with a dilute suspension of *Artemia* sp. nauplii to induce phagocytosis, was pipetted onto each anemone’s oral disc (*n* = ~200 anemones per symbiont species). Anemones were incubated in this symbiont suspension for 24 h before the FSW was changed. All anemones were fed with *Artemia* sp. nauplii three times per week and maintained for 14 weeks at 25°C under 100 μmol photons m^−2^ s^−1^ irradiance on a 12 h:12 h light:dark cycle. Anemones were sampled at weeks 4, 8, and 14 by pooling ∼5 anemones from each container separately and rapidly freezing them in liquid nitrogen, with each pool forming one biological replicate. Anemones were then homogenized using a 5 ml glass tissue homogenizer at 4°C in 2 ml FSW, and symbionts were separated from the host by centrifugation at 500 × *g* for 5 min at 4°C. Total host protein was quantified by analysing the supernatant (i.e. host fraction) with a Qubit® 2.0 Fluorometer and Protein Assay Kit (ThermoFisher Scientific) to calculate symbiont cell density. The symbiont pellet was washed at 4°C with 500 μl FSW, 10 μl were aliquoted for cell counts, and finally the remaining 490 μl were pelleted again by centrifugation at 500 × *g* for 5 min at 4°C. Free-living algal cultures were sampled before inoculation of the hosts and immediately flash-frozen in liquid nitrogen (i.e. Week 0). A total of six biological replicates of free-living algae (*n* = 6) and eight biological replicates at each time-point post-inoculation (*n* = 8, each consisting of algae from ~5 pooled anemones) were taken for analysis, except for *n* = 7 for *D. trenchii* at 14 weeks (Table S6). All samples were kept at −80°C until downstream analysis.

### Protein extraction

Algal pellets were washed with 500 μl of cold HPLC-grade water (Sigma-Aldrich) at 4°C to remove salts and then resuspended in 500 μl 1% sodium deoxycholate (SDC) in HPLC-grade water. The algal suspension was passed five times through a 23-gauge needle (0.337 mm inner diameter) to dislodge any remaining host cell debris (e.g. symbiosome membranes) or protein of host origin from the algal cells. All samples were centrifuged again at 500 × *g* for 5 min at 4°C, and the host-containing supernatant was discarded. For consistency, this process was repeated for Week 0 algae. The algal pellets were resuspended in 500 μl 5% SDC in HPLC-grade water, and the cells were disrupted with an ultrasonic homogenizer (Vibra-Cell™ Ultrasonic VCX 500) and incubated at 85°C for 20 min with 1% β-mercaptoethanol (BME). Residual hydrophobic pigments were removed by ethyl acetate phase transfer [31]. Samples were washed three times with 50 mM Tris pH 8.2 using a 30 kDa molecular mass cut-off filter (14 000*g* × 20 min), resuspended in 400 μl 50 mM Tris pH 8.2 buffer, and digested with 2 μg trypsin overnight at 37°C. Digested peptides were separated by centrifugation through the 30 kDa molecular mass cut-off filter before formic acid (final concentration 1%) was used to terminate trypsin activity and precipitate the remaining SDC. Peptides were then desalted using 50% and 30% acetonitrile (ACN) and resuspended in 0.1% formic acid at 37°C for 30 min, as described in Oakley *et al.* [16]. Symbiont cell densities in anemones hosting *D. trenchii* at Weeks 4 and 8 were insufficient to provide enough symbiont protein to counter even a relatively limited amount of host tissue contamination, so only samples for Week 0 (i.e. prior to inoculation) and Week 14 are reported for this species.

### Liquid chromatography–mass spectrometry

Liquid chromatography-electrospray ionization-tandem mass spectrometry (LC-ESI-MS/MS) was used for peptide analysis. A total of 200 ng of peptides per sample were separated by liquid chromatography (Ultimate 3000, Dionex) on a 15 cm PepMap C18 column (#160321, Thermo Scientific) at 35°C with a non-linear gradient from 96% to 50% buffer A (0.1% formic acid) to buffer B (80% acetonitrile, 0.1% formic acid) and a flow rate of 0.3 μl min^−1^ for 90 min. An Orbitrap Fusion Lumos Tribrid mass spectrometer (Thermo Scientific) was used to analyse peptides ionized by electrospray at 1.8 kV. The orbitrap (resolution 120 000 and scan range 375–1599 m/z) was used for acquiring parent MS spectra. Sequencing was performed via higher energy collisional dissociation fragmentation on the top 20 precursors, and fragment ion spectra were acquired in the linear ion trap with dynamic exclusion enabled for 60 s. Thermo Xcalibur (v4.3) was used to control the instruments. Mass spectrometry data are publicly available at the ProteomeXchange Consortium [32] via the PRIDE [33] repository with identifiers PXD045585 and PXD045587.

### Protein identification and quantification

The Andromeda algorithm in MaxQuant v1.6.17.0 [34] was used to search the resulting tandem MS spectra separately against protein databases constructed from *B. minutum* and *D. trenchii* transcriptomes [35, 36], with standard genetic code for translation. MaxQuant’s default standard contaminant protein database, which contains proteins commonly encountered in laboratory samples (e.g. human keratin and the trypsin experimentally added for protein digestion), was included. These standard laboratory contaminant proteins, when detected, were removed from the list of identified proteins before further analysis. A maximum of two missed cleavages of trypsin digestion, a minimum of two matching peptides per protein, and a minimum peptide length of seven amino acids, all with a false discovery rate of 0.01, were required for peptide and protein validations. This also serves to effectively eliminate the misannotation of remaining Aiptasia proteins as belonging to *B. minutum* or *D. trenchii*. Variable modifications were set as N-terminus acetylation and oxidation of methionine, and fixed modifications were specified as cysteine carbamidomethylation. Matching between runs was enabled with a 0.7 min time window. All spectra from *in hospite* treatments were also searched against Aiptasia protein sequences [37] to confirm that host contamination was minimal.

To further confirm that Aiptasia host proteins were not being erroneously identified as dinoflagellate proteins in our datasets, all *B. minutum* and *D. trenchii* protein sequences reported here were searched by blastp (BLOSUM62, maximum *E*-value 0.05) against a sequence database composed of three dinoflagellate and three cnidarian reference proteomes from UniProt (*Symbiodinium microadriaticum*, UP000186817; *Symbiodinium pilosum*, UP000649617; *Polarella glacialis*, UP000654075; *Exaiptasia diaphana*, UP000887567; *Pocillopora damicornis*, UP000275408; and *Nematostella vectensis*, UP000001593) in Geneious (2022 v1.1, Biomatters Ltd.).

### Statistical analyses

Cell count data were checked for normality by the Shapiro–Wilk test. Homogeneity of variance and covariances were checked by Levene’s test and Box’s M-test, respectively. A mixed two-way analysis of variance (ANOVA) was used to test for differences (*P* < .05) in symbiont density between symbiont species and days, followed by a Bonferroni *post hoc* test to identify specific pairwise differences. All physiological data analyses were conducted in R 4.0.3 [38]. Script and data are accessible via Zenodo (https://zenodo.org/records/10984412).

Bioinformatics software Perseus v.1.6.13.0 [39] was used to discard data from known common contaminant proteins and false identifications. Protein precursor intensities were log_2_-normalized and further used to compare and identify significantly different proteins between treatments. Principal component analysis (PCA) plots were generated with ClustVis [40]. A recently described robust statistical test method, PolyStest integrated with the Miss Test, was used for protein intensity comparisons. The false discovery rate and log-ratio threshold for protein significance were set to ≤0.09 [41]. All detected proteins were searched against the UniProtKB database using DIAMOND [42], with an *E*-value cut-off of ≤1 × 10^−5^. The top manually reviewed SwissProt matches were used to assign protein sequence IDs and annotations, unless no SwissProt matches were detected, where TrEMBL matches were assigned instead. Any unmatched sequences were designated as hypothetical proteins.

## Results and discussion

### Colonization success

Genotyping confirmed the genetic identity of the symbionts, both at the beginning and end of the experiment. The population densities of *B. minutum* and *D. trenchii* were significantly different between the species at all time points (one-way ANOVA, *P* < .05), and by Week 14, *B. minutum* had a 1.5-fold higher population density than *D. trenchii* (5.47 × 10^6^ vs. 3.55 × 10^6^ cells mg^−1^ host protein, respectively) (Supplementary Fig. S1). This pattern is consistent with past studies [43, 44].

### Differentially expressed proteins in B. minutum versus D. trenchii

A total of 1720 and 1536 proteins were detected across all colonization time-points for *B. minutum* and *D. trenchii*, respectively, of which 27 and 21 false matches were discarded. Of these proteins, 336 and 684 were differentially abundant at some point in *B. minutum* and *D. trenchii*, respectively (Supplementary Tables S1–S8). The majority of these proteins (*B. minutum*: 331; *D. trenchii*: 668) were annotated based on homology with sequences in the UniProtKB database (*E*-value ≤ 1 × 10^−5^), and the remaining 21 sequences (5 for *B. minutum* and 16 for *D. trenchii*) were designated as hypothetical proteins. When our reported proteins were searched against a combined dinoflagellate and cnidarian sequence database to confirm that they were of dinoflagellate origin (see Materials and Methods), 97.6% and 97.2% of *B. minutum* and *D. trenchii* proteins, respectively, had a best match against dinoflagellates, 1.4% and 2.2% had a best match against cnidarians, and 0.9% and 0.6% had no match. These results are provided in Table S9.

**Figure 1:**
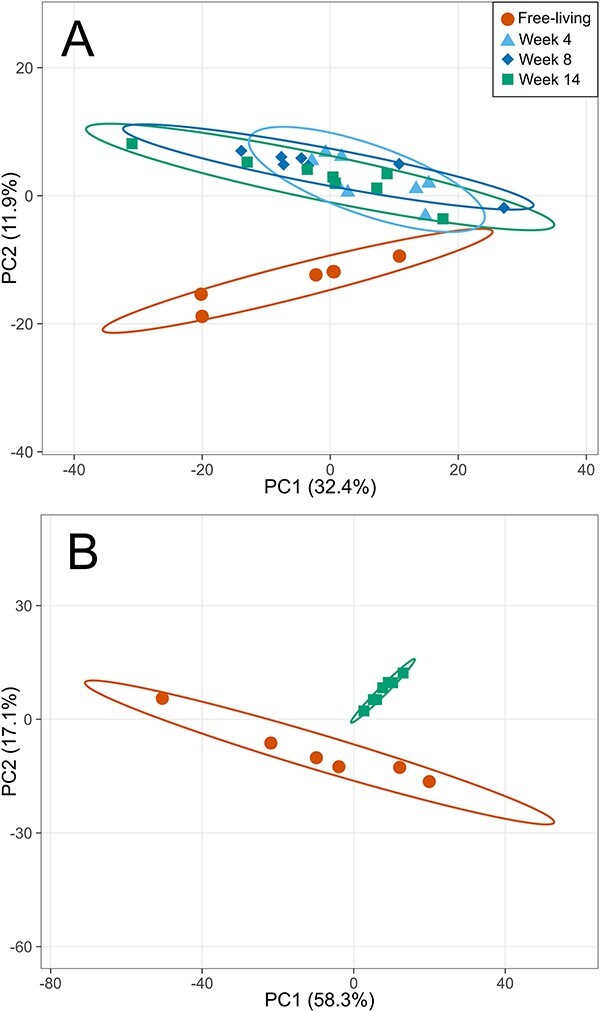
Principal component analysis plots of abundances of all detected symbiont proteins at different times (i.e. free-living, and Weeks 4, 8, and 14 post-inoculation) during colonization of Aiptasia by (A) *Breviolum minutum* and (B) *Durusdinium trenchii*. Biological replicates are grouped by ellipses with 99% confidence intervals.

Generally, the observed proteomes were distinct among the different symbiotic states (i.e. *ex* or *in hospite*), colonization stages, and symbiont species, with the symbiotic state having a major influence irrespective of symbiont species. During host colonization, the abundance of 179 *B. minutum* proteins changed significantly between Weeks 0 and 4; there were fewer changes afterwards, with 54 differentially abundant proteins between Weeks 4 and 8, and only 4 between Weeks 8 and 14. Ultimately, we identified 100 differentially abundant proteins when comparing the start and end of the experiment (i.e. Weeks 0 vs. 14). In comparison, 684 proteins from *D. trenchii* were differentially abundant between Weeks 0 and 14. Similarly, PCA plots showed that the *B. minutum* proteome changed markedly during colonization, with the most distinct profile at week 4 (Fig. 1A). Likewise, the *D. trenchii* proteome at the start and end of the experiment were markedly different (Fig. 1B).

Changes in the *B. minutum* proteome during colonization were consistent with the increasing symbiont population density and modifications likely associated with the persistence of the symbionts inside the host’s cells. In particular, differentially expressed proteins included those with predicted roles in: acquisition of dissolved inorganic carbon (DIC) and photosynthesis; nitrogen metabolism; micro- and macronutrient starvation (e.g. phosphorus); suppression of host immune responses; tolerance of low pH; and management of oxidative stress. All these proteins are discussed below, in terms of the changes observed over the course of colonization by the native symbiont and the differences between the acclimation responses of *B. minutum* relative to the non-native *D. trenchii* (Tables 1 and 2).

**Table 1:** Summarized list of proteins that are differentially abundant (FDR, *q* < 0.05) in *Breviolum minutum* across all time-points during colonization of Aiptasia. Proteins shown here are those discussed in the text. Non-significant comparisons are left blank. The full lists of differentially abundant proteins are available in Supplementary Tables S1–S4.

UniProtKB entry	Protein name	Log_2_ fold change			
		Week 4 vs. free-living	Week 8 vs. 4	Week 14 vs. 8	Week 14 vs. free-living
P42644	14-3-3-like protein GF14 psi		0.43		
A0T0C6	Cytochrome *c*-550	−1.05	0.75		
P00110	Cytochrome *c*6	−1.03	0.92		
A2Y8E0	Ferredoxin—NADP reductase (FNR)	1.96	0.50		2.0
P14070	Flavodoxin	−2.45	1.22		−1.2
P09195	Fructose-1,6-bisphosphatase (FBPase)			−1.23	
Q40297	Fucoxanthin-chlorophyll *a-c* binding protein A	−0.85	0.32		0.6
O09452	Glyceraldehyde-3-phosphate dehydrogenase			−0.66	
Q9STW6	Heat shock 70 kDa protein 6	−1.15	0.79		−0.5
O80796	Membrane-associated protein VIPP1		0.51		
P25776	Oryzain alpha chain			1.46	
A0A1Q9EDY9	Pentatricopeptide repeat-containing protein		0.8		
P51874	Peridinin-chlorophyll *a*-binding protein (PCP)	−2.13	0.72	0.35	
P80483	Peridinin-chlorophyll *a*-binding protein 3 (PCP)	−1.83	0.80		
P26302	Phosphoribulokinase, (PRK)		0.87		0.5
P49481	Photosystem I reaction centre subunit II (PSI-D)	0.50	0.63		1.2
Q7XJ02	Probable L-ascorbate peroxidase 7				1.1
A0A1Q9EFF8	Protein GrpE		0.33		
Q8H0M1	Quinone-oxidoreductase homolog, (ceQORH)		0.44		
P54375	Superoxide dismutase	−2.12	0.91		
A0A1Q9DKM4	Tyrosyl-DNA phosphodiesterase 2		1.03		
P68202	Ubiquitin-40S ribosomal protein S27a		0.67		

**Table 2:** Summarized list of proteins that are differentially abundant (FDR, *q* < 0.05) in *Breviolum minutum* and *Durusdinium trenchii* when comparing free-living cells versus *in hospite* cells after 14 weeks of colonization of Aiptasia. Proteins shown here are those discussed in the text. Non-significant comparisons are left blank. The full lists of differentially abundant proteins between the free-living state (Week 0) and Week 14 of colonization are available in Supplementary Tables S4 and S5.

UniProtKB entry	Protein name	Log_2_ fold change	
		*D. trenchii* Week 14 vs. free-living	*B. minutum* Week 14 vs. free-living
Q5FXM5	Acyl-CoA-binding protein (ACBP)		Unique, *in hospite*
P50430	Arylsulfatase B (ASB)	−2.88	Unique, *in hospite*
A4UHC0	Calmodulin (CaM)	0.36	
Q9I262	Carbonic anhydrase	Unique, free-living	Unique, free-living
Q5BCC5	Carbonic anhydrase	Unique, free-living	
A0A1Q9D9X1	Carbonic anhydrase 2		Unique, free-living
A0A1Q9CS65	Carbonic anhydrase 2	Unique, free-living	Unique, free-living
Q40296	Fucoxanthin-chlorophyll a-c binding protein B, chloroplastic	−1.93	−1.67
Q40300	Fucoxanthin-chlorophyll a-c binding protein F, chloroplastic	−1.57	−1.34
Q59Q30	Glycerophosphoinositol permease 1 (GIT1)		Unique, *in hospite*
B4R9R7	GMP synthase (Glutamine amidotransferase)		Unique, *in hospite*
P62785	Histone H4 variant (TH011)	11.26	
Q7MHB1	Methionine synthase (MS)	Unique, free-living	
P34791	Peptidyl-prolyl cis-trans isomerase (PPIase)	4.30	
Q9LXI4	Purple acid phosphatase 21 (PAP)		Unique, *in hospite*
A0A1Q9F5Z1	Ras-related protein (ORAB-1)		Unique, *in hospite*
P62822	Ras-related protein (Rab-1A)	0.98	
A0A1Q9EXN6	Ras-related protein (Rab-34)	−4.60	−2.99
A6XMY9	S-adenosylmethionine synthase 1 (MAT 1)		1.31
A9NUH8	S-adenosylmethionine synthase 1 (MAT 1)	−1.01	
P20654	Serine/threonine-protein phosphatase (PP1)	Unique, *in hospite*	
O00780	V-type proton ATPase subunit E	Unique, free-living	

### Proteome changes over the course of host colonization

**Figure 2:**
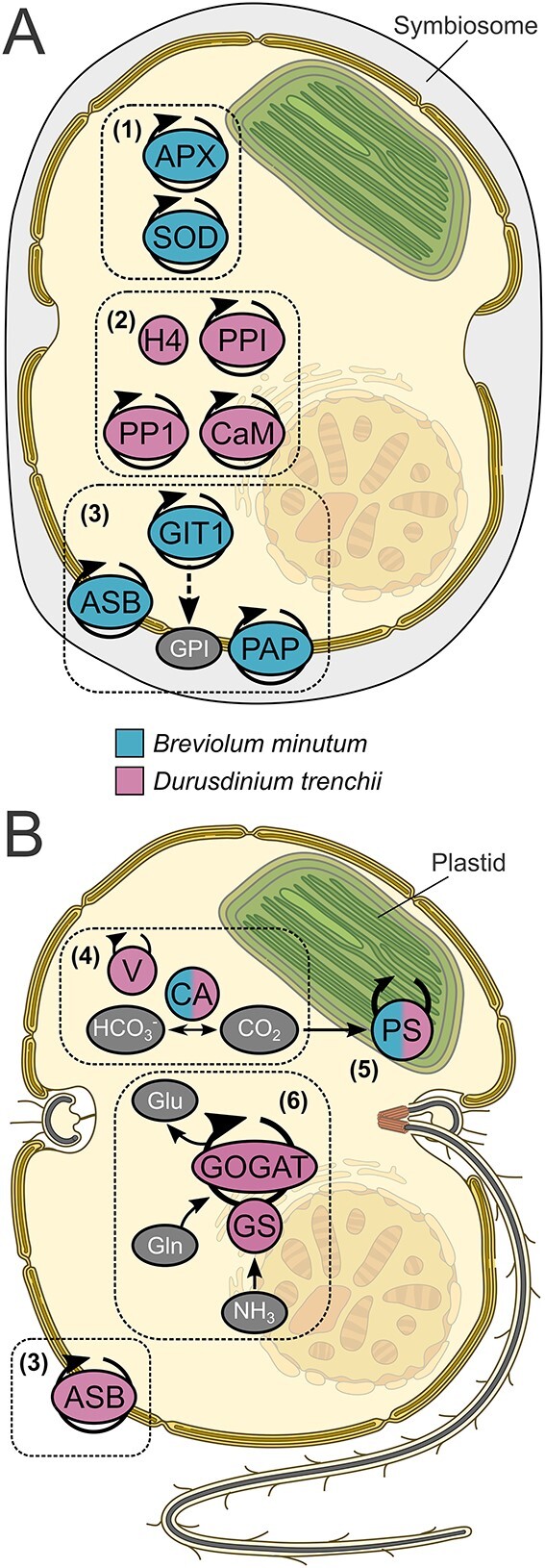
Conceptual diagram summarizing putative symbiont-specific or -regulated cellular processes 14 weeks after colonization of *Exaiptasia diaphana* (A) or while free-living (B), in the native symbiont *Breviolum minutum* and the non-native symbiont *Durusdinium trenchii*. Coloured proteins were significantly more abundant in their respective treatments; e.g. APX was more abundant in *B. minutum* in symbiosis relative to the free-living state. (1) Proteins involved in antioxidant mechanisms, such as APX and SOD, were more abundant only in symbiotic *B. minutum*, potentially as a cellular response for maintaining high rates of photosynthesis and respiration. (2) Peptidyl-prolyl cis-trans isomerase (PPI) is involved in host entry/infection in other systems. Serine/threonine-protein phosphatase (PP1) and calcium/calmodulin (CaM) work dependently and are part of general cell immune pathways, so increased PP1 and CaM in *D. trenchii* may facilitate suppression of the host’s immune system by this non-native symbiont. Histone H4 (H4) suppresses host cell development and immunity in an epigenetic manner. (3) Extracellular and cell surface proteins. GIT1 was only found in symbiotic *B. minutum*, where it may prevent host autophagy, and facilitate inter-partner communication and cell recognition. Extracellular purple acid phosphatase (PAP), which plays a role in phosphate uptake, was only found in symbiotic *B. minutum*. ASB is involved in autophagy and was upregulated in free-living *D. trenchii* and symbiotic *B. minutum*. (4) *B. minutum* and *D*. *trenchii* CA were upregulated when free-living. Vacuolar proton pumps (V), which facilitate CA activity, were only upregulated in free-living *D*. *trenchii*. CA converts bicarbonate ions (HCO_3_^−^) to carbon dioxide (CO_2_), which can be further used for photosynthesis. Upregulation of algal CAs and V-type ATPase in the free-living state might be a response to a lack of readily available CO_2_ provided by host CAs and proton pumps when *in hospite*. (5) Photosystem (PS) I and II proteins involved in light harvesting, as well as chloroplastic electron transport proteins and chloroplastic maintenance upregulation in *B. minutum* and *D. trenchii*. (6) Ammonium is assimilated via the glutamine synthetase/glutamine 2-oxoglutarate amidotransferase (GS/GOGAT) pathway, though this pathway was upregulated only in free-living *D. trenchii.* Dinoflagellate image modified from Keeling and Eglit [99].

Nearly one-third (20 out of 57) *B. minutum* proteins that were differentially abundant during colonization (i.e. Week 4 vs. 8 and Week 8 vs. 14) were putatively linked to photosynthesis (Fig. 2). Homologues of light-harvesting proteins of Photosystems I and II, such as chlorophyll *a*-chlorophyll *c*_2_-peridinin protein (apcPC, misannotated as “fucoxanthin-chlorophyll *a-c* binding protein” in UniProt; see [77]), and peridinin-chlorophyll *a*-binding protein (PCP), the major light-harvesting proteins of the Symbiodiniaceae, were identified in *B. minutum* across all time-points [45]. In both *B. minutum* and *D. trenchii*, abundances of apcPC and PCP were broadly reduced in symbiosis at week 14 relative to the free-living state (Tables 1 and 2). Both apcPCs and PCPs in *B. minutum* were upregulated at Week 8 vs. Week 4, peaking at the same time as the symbiont population density (Supplementary Fig. S1). An increase in pigmentation due to symbiont self-shading is a light-adaptive strategy, so the observed patterns were unsurprising [46, 47]. This increase in light-harvesting proteins was coupled with an upregulation of chloroplastic electron transport proteins, such as cytochromes, flavodoxin, PSI reaction centre proteins, and ferredoxin-NADP reductase, as well as proteins associated with the reductive pentose-phosphate cycle, such as phosphoribulokinase, which provides the substrate for Rubisco [48] (Table 1). Similarly, proteins involved in translation, protein folding, chloroplast maintenance, and photosystem repair were more abundant at Week 8.

A homologue of 14-3-3-protein (GF14), which is involved in the response to cellular nitrogen starvation by controlling glutamine synthetase and nitrate reductase via phosphorylation [28], was more abundant at Week 8. This coincided with the maximum population density of *B. minutum*, and so it might be linked to the host’s need to regulate symbiont proliferation via nitrogen limitation. Two Calvin cycle enzymes, glyceraldehyde-3-phosphate dehydrogenase and fructose-1,6-bisphosphatase, were less abundant at Week 14 vs. Week 8, as was PCP (Table S3). These may reflect delayed cell acclimation to greater self-shading and a reduced rate of carbon fixation per cell in the steady-state symbiosis, which showed little change in either cell density or proteomic profile between Weeks 8 and 14 (Table S3).

### Comparative responses of B. minutum versus D. trenchii

#### Oxidative stress

Homologues of algal antioxidant network proteins were identified in both *B. minutum* and *D. trenchii*. Oxidants, including reactive oxygen species (ROS), can damage DNA, proteins, and cell membranes [49]. The dinoflagellate symbiont’s antioxidant network, in response to increased oxidants under high temperature and irradiance has been characterized [50, 51], though ROS are also regular by-products of respiration, photosynthesis, and photorespiration; antioxidant defences are therefore needed at all times. Here, homologues of symbiont superoxide dismutase (SOD) and ascorbate peroxidase (APX), which work together to detoxify superoxide and hydrogen peroxide, were detected in *B. minutum* at all time points, with both increasing progressively during host colonization (Table 1). This is unsurprising, as photosynthetic activity by the symbionts can rapidly induce a state of hyperoxia in the host’s tissues [52]. Homologues of APX and SOD, as well as other antioxidant network proteins, such as glutathione reductase and cytochrome *c* peroxidase, were also detected in *D. trenchii*; however, contrary to the results for *B. minutum*, these proteins were downregulated when *in hospite* (Table S5). The reasons for this are unclear, but this was coupled with a downregulation of proteins involved in photosynthesis. *D. trenchii* has been previously reported to have reduced photosynthetic incorporation of ^13^C per cell in comparison to *B. minutum* when in symbiosis with Aiptasia [53]. Therefore, downregulation of the antioxidant network in this species might be associated with less photosynthesis.

#### Dissolved inorganic carbon transport

Three and 16 homologues of symbiont carbonic anhydrases (CAs) were upregulated in free-living *B. minutum* and *D. trenchii*, respectively (Fig. 2). All three CA homologues from *B. minutum* were exclusive to the free-living state, as well as 12 CA homologues from *D. trenchii* (Table 2). DIC is crucial for both photosynthesis and host calcification in corals, although DIC in ambient seawater is mostly present as bicarbonate [HCO_3_^−^], which must be converted to carbon dioxide [CO_2_] to freely pass through cell membranes. Therefore, the cnidarian host appears to actively promote DIC uptake via carbon concentrating mechanisms (CCMs), which facilitate the interconversion of CO_2_ and HCO_3_^−^ (the final equilibrium depends on the pH in the vicinity of the enzyme) and delivery of inorganic carbon to the algal cell [54, 55]. Proton pumps are also critical for CCMs, whereas acidification in the region of the CA drives the conversion from HCO_3_^−^ to CO_2_, which is the specific substrate for Rubisco [56]. Cnidarian host CAs and proton pumps significantly accumulate upon symbiosis establishment, which is likely required for supplying the symbionts with DIC [14, 16]. Dinoflagellate genes encoding proton pumps in cnidarian- and bivalve-dinoflagellate symbioses also form CCMs [57, 58]; however, homologues of proton pumps, like V-type proton ATPase, were only detected here in free-living *D. trenchii*. Upregulated algal CAs and proton pumps of the algae in the free-living state might be a response to low CO_2_ availability, which can be facilitated by host CAs and proton pumps [14, 56].

#### Nitrogen metabolism

A group of proteins predicted to function in the uptake and metabolism of dissolved inorganic nitrogen (DIN) [54] were identified in both Symbiodiniaceae species at both the beginning and end of the experiment (Fig. 2). Ammonium (NH_4_^+^) and nitrate (NO_3_^−^) are the most available forms of DIN in seawater, with ammonium being preferred over nitrate by symbiotic cnidarians [59]. Both partners can assimilate DIN; however, the majority is assimilated by the symbionts via the glutamine synthetase/glutamine 2-oxoglutarate amidotransferase (GS/GOGAT) pathway. This pathway generates the amino acid glutamate, which can also serve as a precursor for other amino acids and purines/ureides. The algal symbionts also assimilate ammonium via glutamate dehydrogenase (GDH), although this has a lower affinity for ammonium than does GS/GOGAT [60, 61]. In the present study, homologues of GS/GOGAT were isolated from both symbiont species, although a homologue of GDH was only detected in symbiotic *B. minutum*. However, in *B. minutum*, these enzymes were not differentially abundant when either free-living or *in hospite*, or between the different time-points during colonization. In contrast, in *D. trenchii*, four and two homologues of GS and GOGAT, respectively, were downregulated *in hospite* relative to the free-living state. Nitrogen limitation has been suggested as the primary mechanism by which the host controls the proliferation of its symbionts [62, 63], so the lower abundance of GS/GOGAT in symbiotic *D. trenchii* might reflect greater *N* availability *in hospite*, due to host-derived ammonium, relative to the free-living state. The reasons for differences between symbiont species are unknown, but previous evidence suggests that symbiont *N* access is more tightly regulated in Aiptasia colonized by *B. minutum* rather than *D. trenchii* [22, 53], and that *D. trenchii* may manipulate host metabolism to enhance ammonium availability via the urea cycle [19, 22], reducing the need for GS/GOGAT activity. Differences in *N* assimilation pathways may also reflect the somewhat lower symbiont densities in Aiptasia hosting *D. trenchii*.

Regardless of the assimilatory mechanism, nitrogen is used as a precursor for the *de novo* synthesis of amino acids and nucleotides [64]. A group of proteins involved in methionine synthesis and metabolism were found in both algal species. Methionine synthesis is of interest in this symbiosis, as some symbiotic cnidarians (e.g. *Acropora* corals) appear to lack complete methionine synthesis pathways and may acquire translocated methionine, or necessary intermediates, from their dinoflagellate symbionts with complementary synthesis pathways [65]; Aiptasia may possess an entire methionine synthesis pathway [62]. Here, *D. trenchii* increased accumulation of methionine and S-adenosylmethionine synthase in the free-living state, whereas *B. minutum* showed upregulation of S-adenosylmethionine synthase when in symbiosis (Table 2); the latter contributes to methionine recycling [66]. This pattern could help to explain the observation of Sproles *et al.* [14] and Matthews *et al.* [19], who noted that Aiptasia likely receives more methionine from *B. minutum* than *D. trenchii*, based on proteomic and metabolomic evidence [12, 19]. Additionally, a homologue of guanosine monophosphate synthetase (GMP), with a predicted function in the *de novo* synthesis of guanine nucleotides by hydrolyzing glutamine [67], was found to be elevated in *B. minutum* at Week 14. The alternative fate of glutamine in the symbionts is its storage in high-nitrogen compounds synthesized via the pathway involving purine biosynthesis [61]. Therefore, elevated levels of GMP in symbiotic *B. minutum* in contrast to free-living and *in hospite D. trenchii*, suggest better metabolic integration of the native symbionts with their host than with the more opportunistic non-native *D. trenchii* [43, 53].

#### Requirements for living inside the symbiosome

In cnidarian–dinoflagellate associations, the endosymbionts exist in a host-derived late arrested phagosome, the “symbiosome” [68]. Inter-partner communication occurs across the symbiosome membrane, as described in detail by Rosset *et al.* [69], and the symbionts must cope with the environmental conditions imposed by this structural containment. In the current study, extracellular and cell surface proteins of the algae were only abundant in the symbiotic state (Fig. 2). For instance, a homologue of acyl coenzyme A binding protein (ACBP) was consistently found from Week 4 onwards only in *B. minutum*. ACBP’s role in intracellular lipid trafficking is well described [70]. However, the ACBP homologue detected here is putatively located at the extracellular matrix (ECM) (e.g. cell wall), with predicted functions in impacting cell morphogenesis, structural support and protection, differentiation, and signalling [70]. Extracellular ACBPs are increasingly produced under abiotic stress, including nitrogen limitation and low pH [71, 72], both of which are characteristic of the conditions experienced inside the symbiosome [62, 73] and hence may contribute to the increased accumulation of this protein that we observed *in hospite*.

A homologue of the extracellular purple acid phosphatase (PAP) was exclusively present in *B. minutum in hospite*, although it was similarly abundant across all colonization time-points. Extracellular PAPs play a role in inorganic phosphate (Pi) acquisition and scavenging during phosphorus starvation by hydrolyzing Pi from organophosphate compounds, which frees it for uptake and assimilation, as previously reported for both plant-microbe symbioses and free-living marine diatoms [74, 75]. Severe Pi limitation in symbiosis may induce bleaching by restricting symbiont cell division [76, 77]. PAPs have also previously been localized to the symbiosome surface in both plant-microbe symbioses and the cnidarian–dinoflagellate symbiosis [78, 79]. Additionally, two acid phosphatases involved in carrier-mediated Pi transportation have been reported in the coral *Acropora formosa*; one is external to the algal plasmalemma and involved in phosphate ester translocation from the cytoplasm of the cnidarian cells into the symbiosome [80]. Furthermore, the abundance of two homologues of glycerophosphoinositol permease 1 (GIT1) progressively increased in *B. minutum* during colonization, being most abundant at Week 14. GIT1 facilitates the transport of glycerophosphoinositol (GPI), a product of phosphatidylinositol deacylation, across the cell membrane. GPI can be used as a source of Pi, especially during phosphorus starvation or in acidic environments [81, 82], both of which are likely features of the intra-symbiosome environment [73, 80]. Neither of these proteins (i.e. PAP and GIT1) were detected in *D. trenchii*, suggesting either that this symbiont species uses a different mechanism for sourcing Pi or, once again, that host-symbiont cellular integration is sub-optimal in this association [19, 44].

Another cell surface protein detected was a homologue of arylsulfatase B (ASB), which has a predicted function in autophagy and metabolization of sulphated polysaccharides (Fig. 2) [83, 84]. ASB was not observed in free-living *B. minutum* but increased during colonization, with its maximum abundance at Week 14. In marked contrast, in *D. trenchii*, ASB was detected both while free-living and after 14 weeks in symbiosis, although it was more abundant during free-living growth. Levels of this protein are reduced in the larvae of the coral *Porites astreoides*, potentially in response to low symbiont numbers and the need for the symbionts to proliferate [85]. Whether ASB detected in the two algal species here is similarly involved with autophagy is unknown, though the coordination of host-symbiont biomass is important for the maintenance of the association [7]. Alternatively, given the high abundance of ASB in free-living *D. trenchii*, it is plausible that the symbionts employ ASB in the degradation of sulphated polysaccharides, which would allow their use for heterotrophic growth [53, 86].

A group of homologues of Ras-associated binding proteins (e.g. RAB1 and RAB34) were differentially abundant *ex* and *in hospite* in both *B. minutum* and *D. trenchii*. *Cnidarian Rab* family proteins are known to increase after symbiont inoculation and are thought to be responsible for symbiosome formation via endocytosis and symbiont density regulation via exocytosis and/or phagocytosis [87–89]. Rab proteins have also been reported in the symbionts, again with proposed roles in exocytosis [26] and manipulation of the symbiosome membrane [90]*.* In the current study, homologues of Rab34 from *B. minutum* and *D. trenchii* were more abundant while free-living than *in hospite* (2.99-fold and 4.6-fold, respectively), potentially due to endocytosis of extracellular particles via heterotrophy when the alga is free-living [86, 91]. In contrast, Rab1 homologues were elevated in both species at Week 14 after colonization (Table 2), although this increase was much greater in *B. minutum* than in *D. trenchii* and coincided with a decline in the *B. minutum* population (the *D. trenchii* population was still growing at this stage) (Supplementary Fig. S1). However, the exact role of Rab1 in the regulation of symbiont growth/biomass remains unclear.

#### Immunosuppression

Most of the differentially abundant proteins isolated from *D. trenchii* when *in hospite* had predicted functions associated with host cell immunosuppression, whereas none of these proteins were differentially abundant in *B. minutum* (Fig. 2). For example, a homologue of peptidyl-prolyl cis-trans isomerase (PPIase) was 4.3-fold more abundant at Week 14 in *D. trenchii*. PPIase activity is thought to facilitate the proper folding of specific proteins [92]; however, PPIases can also play an important role in host-microbe associations, such as host entry/infection [93]. In the cnidarian–dinoflagellate symbiosis, dose-dependent inhibition of host PPIase causes symbiont expulsion from Aiptasia [92], so it is conceivable that elevation of PPIase in symbiotic *D. trenchii* reflects an antagonistic reaction to potential expulsion from the host. In addition, a homologue of serine/threonine-protein phosphatase (PP1) was exclusively identified in *D. trenchii* when *in hospite*. Serine/threonine-protein phosphatases/kinases, which depend on calcium/calmodulin (CaM)-mediated signalling, are important in cell proliferation and signalling. Moreover, CaM-dependent PP1 activity is involved in immunity, and PP1 levels are elevated during the establishment of other symbioses [94]. Furthermore, inhibition of PP1 in Aiptasia is associated with oxidative stress and symbiont expulsion [92], whereas increased transcriptional regulation of PP1 occurs in apicomplexan parasites (*Plasmodium* spp.) upon host infection [95]. A homologue of CaM increased in this current study in parallel with PP1, consistent with a degree of functional co-dependency. Symbiodiniacean PP1 is transcriptionally upregulated under different environmental stresses, such as low phosphorus availability and pH, and increased temperature [96–98]. Here, its increased abundance may participate in the suppression of the host immune response.

## Conclusion

This study identified changes in the proteome of *B. minutum* during colonization of its native host, the model cnidarian Aiptasia. In particular, proteins involved in photosynthesis/respiration, nutrient exchange, oxidative stress, and cell apoptosis became more abundant as colonization progressed, most likely linked to increasing symbiont cell density, lower amounts of available nutrients, and self-shading. This study also compared the proteomes of *B. minutum* and the non-native *D. trenchii* after full colonization of Aiptasia. The most distinct differences between these symbiont species related to cell surface proteins, some of which were not detectable in *D. trenchii*. Moreover, *B. minutum* exhibited increased accumulation of proteins associated with nutrient limitation (consistent with host-regulated nutrient supply), whereas the majority of elevated proteins in *D. trenchii* have potential roles in host immunosuppression. Taken together, these observations are consistent with the view that *B. minutum* forms a functionally well-integrated symbiosis with Aiptasia, whereas *D. trenchii* does not, reflecting its reputation as a more metabolically costly opportunist [12, 14, 19, 22, 44]. The acquisition of more thermally tolerant symbionts, such as *D. trenchii*, has been suggested as an adaptive strategy under scenarios of global warming. However, our study adds to the increasing body of evidence that host-symbiont specificity, and its consequences on symbiotic integration and holobiont function will limit such opportunities [43]. Further research is needed to determine whether inter-partner integration and function in novel symbioses can improve over time and in response to thermal acclimation, which would augment our knowledge and allow for better predictions of the responses of coral reefs to climate change.

## Supplementary Material

Supplementary_Tables_S1-S9_Rev2d_wrae122

Mashini_et_al_Supp_Figure_1_wrae122

Mashini_et_al_Supp_Figure_1_Legend_wrae122

## Data Availability

Mass spectrometry data are publicly available at the ProteomeXchange Consortium [32] via the PRIDE [33] repository with identifiers PXD045585 and PXD045587.

## Additional information

Correspondence and requests for materials, including cultured Aiptasia, should be addressed to Simon K. Davy.

## References

[1] Veron JEN , Hoegh-Guldberg O, Lenton TM et al. The coral reef crisis: the critical importance of <350 ppm CO_2_. Mar Pollut Bull 2009;58:1428–36. 10.1016/j.marpolbul.2009.09.009 19782832

[2] Muscatine L , Porter JW. Reef corals: mutualistic symbioses adapted to nutrient-poor environments. Bioscience 1977;27:454–60. 10.2307/1297526

[3] LaJeunesse TC , Parkinson JE, Gabrielson PW et al. Systematic revision of Symbiodiniaceae highlights the antiquity and diversity of coral endosymbionts. Curr Biol 2018;28:2570–2580.e6. 10.1016/j.cub.2018.07.008 30100341

[4] Nitschke MR , Craveiro SC, Brandão C et al. Description of *Freudenthalidium* gen. nov. and *Halluxium* gen. nov. to formally recognize clades Fr3 and H as genera in the family Symbiodiniaceae (Dinophyceae). J Phycol 2020;56:923–40. 10.1111/jpy.12999 32267533

[5] Pochon X , LaJeunesse TC. *Miliolidium* n. gen, a new symbiodiniacean genus whose members associate with soritid foraminifera or are free-living. J Eukaryot Microbiol 2021;68:1–9. 10.1111/jeu.12856 33966311

[6] Rowan R , Knowlton N, Baker A et al. Landscape ecology of algal symbionts creates variation in episodes of coral bleaching. Nature 1997;388:265–9. 10.1038/40843 9230434

[7] Davy SK , Allemand D, Weis VM. Cell biology of cnidarian-dinoflagellate symbiosis. Microbiol Mol Biol Rev 2012;76:229–61. 10.1128/MMBR.05014-11 22688813 PMC3372257

[8] Bay LK , Cumbo VR, Abrego D et al. Infection dynamics vary between *Symbiodinium* types and cell surface treatments during establishment of endosymbiosis with coral larvae. Diversity 2011;3:356–74. 10.3390/d3030356

[9] Hughes TP , Kerry JT, Álvarez-Noriega M et al. Global warming and recurrent mass bleaching of corals. Nature 2017;543:373–7. 10.1038/nature21707 28300113

[10] LaJeunesse TC , Wham DC, Pettay DT et al. Ecologically differentiated stress-tolerant endosymbionts in the dinoflagellate genus *Symbiodinium* (Dinophyceae) Clade D are different species. Phycologia 2014;53:305–19. 10.2216/13-186.1

[11] Hume BCC , D’Angelo C, Smith EG et al. *Symbiodinium thermophilum* sp. nov., a thermotolerant symbiotic alga prevalent in corals of the world’s hottest sea, the Persian/Arabian Gulf. Sci Rep 2015;5:8562. 10.1038/srep08562 25720577 PMC4342558

[12] Pettay D , Whama DC, Smith RT et al. Microbial invasion of the Caribbean by an Indo-Pacific coral zooxanthella. Proc Natl Acad Sci U S A 2015;112:7513–8. 10.1073/pnas.1502283112 26034268 PMC4475936

[13] Little AF , van Oppen MJH, Willis BL. Flexibility in algal endosymbioses shapes growth in reef corals. Science 2004;304:1492–4. 10.1126/science.1095733 15178799

[14] Sproles AE , Oakley CA, Matthews JL et al. Proteomics quantifies protein expression changes in a model cnidarian colonised by a thermally tolerant but suboptimal symbiont. ISME J 2019;13:2334–45. 10.1038/s41396-019-0437-5 31118473 PMC6775970

[15] Oakley CA , Durand E, Wilkinson SP et al. Thermal shock induces host proteostasis disruption and endoplasmic reticulum stress in the model symbiotic cnidarian Aiptasia. J Proteome Res 2017;16:2121–34. 10.1021/acs.jproteome.6b00797 28474894

[16] Oakley CA , Ameismeier MF, Peng L et al. Symbiosis induces widespread changes in the proteome of the model cnidarian Aiptasia. Cell Microbiol 2016;18:1009–23. 10.1111/cmi.12564 26716757

[17] Lehnert EM , Mouchka ME, Burriesci MS et al. Extensive differences in gene expression between symbiotic and aposymbiotic cnidarians. Genes Genomes Genet 2014;4:277–95. 10.1534/g3.113.009084 PMC3931562 24368779

[18] Lehnert EM , Burriesci MS, Pringle JR. Developing the anemone Aiptasia as a tractable model for cnidarian-dinoflagellate symbiosis: the transcriptome of aposymbiotic *A. pallida*. BMC Genomics 2012;13:1–10. 22726260 10.1186/1471-2164-13-271 PMC3427133

[19] Matthews JL , Crowder CM, Oakley CA et al. Optimal nutrient exchange and immune responses operate in partner specificity in the cnidarian-dinoflagellate symbiosis. Proc Natl Acad Sci U S A 2017;114:13194–9. 10.1073/pnas.1710733114 29158383 PMC5740609

[20] Weis VM , Davy SK, Hoegh-Guldberg O et al. Cell biology in model systems as the key to understanding corals. Trends Ecol Evol 2008;23:369–76. 10.1016/j.tree.2008.03.004 18501991

[21] Thornhill DJ , Xiang Y, Pettay DT et al. Population genetic data of a model symbiotic cnidarian system reveal remarkable symbiotic specificity and vectored introductions across ocean basins. Mol Ecol 2013;22:4499–515. 10.1111/mec.12416 23980764

[22] Matthews JL , Oakley CA, Lutz A et al. Partner switching and metabolic flux in a model cnidarian–dinoflagellate symbiosis. Proc R Soc B 2018;285:20182336. 10.1098/rspb.2018.2336 PMC6283946 30487315

[23] Pasaribu B , Weng LC, Lin IP et al. Morphological variability and distinct protein profiles of cultured and endosymbiotic *Symbiodinium* cells isolated from *Exaiptasia pulchella*. Sci Rep 2015;5:15353. 10.1038/srep15353 26481560 PMC4611179

[24] Maor-Landaw K , van Oppen MJH, McFadden GI. Symbiotic lifestyle triggers drastic changes in the gene expression of the algal endosymbiont *Breviolum minutum* (Symbiodiniaceae). Ecol Evol 2020;10:451–66. 10.1002/ece3.5910 31993121 PMC6972872

[25] Bellantuono AJ , Dougan KE, Granados-Cifuentes C et al. Free-living and symbiotic lifestyles of a thermotolerant coral endosymbiont display profoundly distinct transcriptomes under both stable and heat stress conditions. Mol Ecol 2019;28:5265–81. 10.1111/mec.15300 31693775

[26] Weston AJ , Dunlap WC, Shick JM et al. A profile of an endosymbiont-enriched fraction of the coral *Stylophora pistillata* reveals proteins relevant to microbial-host interactions. Mol Cell Proteomic*s* 2012;11:M111.015487–7. 10.1074/mcp.M111.015487 PMC3433924 22351649

[27] Tsang Min Ching SJ , Chan WY, Perez-Gonzalez A et al. Colonization and metabolite profiles of homologous, heterologous and experimentally evolved algal symbionts in the sea anemone *Exaiptasia diaphana*. ISME Commun 2022;2:30. 10.1038/s43705-022-00114-7 37938648 PMC9723793

[28] Xiang T , Lehnert E, Jinkerson RE et al. Symbiont population control by host-symbiont metabolic interaction in Symbiodiniaceae-cnidarian associations. Nat Commun 2020;11:108. 10.1038/s41467-019-13963-z 31913264 PMC6949306

[29] Mashini AG , Oakley CA, Beepat SS et al. The influence of symbiosis on the proteome of the *Exaiptasia* endosymbiont *Breviolum minutum*. Microorganisms 2023;11:292. 10.3390/microorganisms11020292 36838257 PMC9967746

[30] Matthews JL , Sproles AE, Oakley CA et al. Menthol-induced bleaching rapidly and effectively provides experimental aposymbiotic sea anemones (Aiptasia sp.) for symbiosis investigations. J Exp Biol 2016;219:306–10. 26596538 10.1242/jeb.128934

[31] Yeung Y , Stanley ER. Rapid detergent removal from peptide samples with ethyl acetate for mass spectrometry analysis. Curr Protoc Protein Sci 2010;59:12–6. 10.1002/0471140864.ps1612s59 PMC2852680 20155730

[32] Deutsch EW , Csordas A, Sun Z et al. The ProteomeXchange consortium in 2017: supporting the cultural change in proteomics public data deposition. Nucleic Acids Res 2017;45:D1100–6. 10.1093/nar/gkw936 27924013 PMC5210636

[33] Perez-Riverol Y , Csordas A, Bai J et al. The PRIDE database and related tools and resources in 2019: improving support for quantification data. Nucleic Acids Res 2019;47:D442–50. 10.1093/nar/gky1106 30395289 PMC6323896

[34] Cox J , Hein MY, Luber CA et al. Accurate proteome-wide label-free quantification by delayed normalization and maximal peptide ratio extraction, termed MaxLFQ. Mol Cell Proteomics 2014;13:2513–26. 10.1074/mcp.M113.031591 24942700 PMC4159666

[35] Parkinson JE , Baumgarten S, Michell CT et al. Gene expression variation resolves species and individual strains among coral-associated dinoflagellates within the genus *Symbiodinium*. Genome Biol Evol 2016;8:665–80. 10.1093/gbe/evw019 26868597 PMC4824173

[36] Camp EF , Kahlke T, Signal B et al. Proteome metabolome and transcriptome data for three Symbiodiniaceae under ambient and heat stress conditions. Sci Data 2022;9:153. 10.1038/s41597-022-01258-w 35383179 PMC8983644

[37] Baumgarten S , Simakov O, Esherick LY et al. The genome of Aiptasia, a sea anemone model for coral symbiosis. Proc Natl Acad Sci U S A 2015;112:11893–8. 10.1073/pnas.1513318112 26324906 PMC4586855

[38] R Core Team . R: A Language and Environment for Statistical Computing. Vienna, Austria: R Foundation for Statistical Computing, 2020.

[39] Tyanova S , Cox J. Perseus: a bioinformatics platform for integrative analysis of proteomics data in cancer research. Methods Mol Biol 2018;1711:133–48. 29344888 10.1007/978-1-4939-7493-1_7

[40] Metsalu T , Vilo J. ClustVis: a web tool for visualizing clustering of multivariate data using principal component analysis and heatmap. Nucleic Acids Res 2015;43:W566–70. 10.1093/nar/gkv468 25969447 PMC4489295

[41] Schwämmle V , Hagensen CE, Rogowska-Wrzesinska A et al. PolySTest: robust statistical testing of proteomics data with missing values improves detection of biologically relevant features. Mol Cell Proteomics 2020;19:1396–408. 10.1074/mcp.RA119.001777 32424025 PMC8015005

[42] Buchfink B , Xie C, Huson DH. Fast and sensitive protein alignment using DIAMOND. Nat Methods 2014;12:59–60. 10.1038/nmeth.3176 25402007

[43] Gabay Y , Parkinson JE, Wilkinson SP et al. Inter-partner specificity limits the acquisition of thermotolerant symbionts in a model cnidarian-dinoflagellate symbiosis. ISME J 2019;13:2489–99. 10.1038/s41396-019-0429-5 31186513 PMC6776018

[44] Gabay Y , Weis VM, Davy SK. Symbiont identity influences patterns of symbiosis establishment, host growth, and asexual reproduction in a model cnidarian-dinoflagellate symbiosis. Biol Bull 2018;234:1–10. 10.1086/696365 29694802

[45] Boldt L , Yellowlees D, Leggat W. Hyperdiversity of genes encoding integral light-harvesting proteins in the dinoflagellate *Symbiodinium* sp. PLoS One 2012;7:1–13. 10.1371/journal.pone.0047456 PMC3480386 23112815

[46] Kaiser P , Schlichter D, Fricke HW. Influence of light on algal symbionts of the deep water coral *Leptoseris fragilis*. Mar Biol 1993;117:45–52. 10.1007/BF00346424

[47] Kahng SE , Garcia-Sais JR, Spalding HL et al. Community ecology of mesophotic coral reef ecosystems. Coral Reefs 2010;29:255–75. 10.1007/s00338-010-0593-6

[48] Nickelsen K . The path of carbon in photosynthesis (1937–1954). In: History, Philosophy and Theory of the Life Sciences. Springer, Dordrecht, 2015, 201–50.

[49] Downs CA , Fauth JE, Halas JC et al. Oxidative stress and seasonal coral bleaching. Free Radic Biol Med 2002;33:533–43. 10.1016/S0891-5849(02)00907-3 12160935

[50] Wietheger A , Starzak DE, Gould KS et al. Differential ROS generation in response to stress in *Symbiodinium* spp. Biol Bull 2018;234:11–21. 10.1086/696977 29694799

[51] Krueger T , Becker S, Pontasch S et al. Antioxidant plasticity and thermal sensitivity in four types of *Symbiodinium* sp. J Phycol 2014;50:1035–47. 10.1111/jpy.12232 26988785

[52] Richier S , Merle PL, Furla P et al. Characterization of superoxide dismutases in anoxia- and hyperoxia-tolerant symbiotic cnidarians. Biochim Biophys Acta 2003;1621:84–91. 10.1016/S0304-4165(03)00049-7 12667614

[53] Sproles AE , Oakley CA, Krueger T et al. Sub-cellular imaging shows reduced photosynthetic carbon and increased nitrogen assimilation by the non-native endosymbiont *Durusdinium trenchii* in the model cnidarian Aiptasia. Environ Microbiol 2020;22:3741–53. 10.1111/1462-2920.15142 32592285

[54] Furla P , Galgani I, Durand I et al. Sources and mechanisms of inorganic carbon transport for coral calcification and photosynthesis. J Exp Biol 2000;203:3445–57. 10.1242/jeb.203.22.3445 11044383

[55] Allemand D , Furla P, Bénazet-Tambutté S. Mechanisms of carbon acquisition for endosymbiont photosynthesis in *Anthozoa*. Can J Bot 1998;76:925–41.

[56] Furla P , Allemand D, Orsenigo MN. Involvement of H^+^-ATPase and carbonic anhydrase in inorganic carbon uptake for endosymbiont photosynthesis. Am J Physiol Regul Integr Comp Physiol 2000;278:R870–81. 10.1152/ajpregu.2000.278.4.R870 10749774

[57] Bertucci A , Tambutté É, Tambutté S et al. Symbiosis-dependent gene expression in coral-dinoflagellate association: cloning and characterization of a P-type H^+^-ATPase gene. Proc R Soc B 2010;277:87–95. 10.1098/rspb.2009.1266 PMC2842621 19793745

[58] Mies M , Voolstra CR, Castro CB et al. Expression of a symbiosis-specific gene in *Symbiodinium* type A1 associated with coral, nudibranch and giant clam larvae. R Soc Open Sci 2017;4:170253. 10.1098/rsos.170253 28573035 PMC5451836

[59] Grover R , Maguer JFF, Allemand D et al. Uptake of dissolved free amino acids by the scleractinian coral *Stylophora pistillata*. J Exp Biol 2008;211:860–5. 10.1242/jeb.012807 18310111

[60] Roberts JM , Fixter LM, Davies PS. Ammonium metabolism in the symbiotic sea anemone *Anemonia viridis*. Hydrobiologia 2001;461:25–35. 10.1023/A:1012752828587

[61] Pernice M , Meibom A, Van Den Heuvel A et al. A single-cell view of ammonium assimilation in coral-dinoflagellate symbiosis microbe-microbe and microbe-host interactions. ISME J 2012;6:1314–24. 10.1038/ismej.2011.196 22222466 PMC3379633

[62] Wang JT , Douglas AE. Essential amino acid synthesis and nitrogen recycling in an alga-invertebrate symbiosis. Mar Biol 1999;135:219–22. 10.1007/s002270050619

[63] Yellowlees D , Rees T, Fitt W. Effect of ammonium-supplemented seawater on glutamine synthetase and glutamate dehydrogenase activities in host tissue and *zooxanthellae* of *Pocillopora damicornis* and on ammonium uptake rates of the *zooxanthellae*. Pac Sci 1994;48:291–5.

[64] Newsholme P , Procopio J, Ramos Lima MM et al. Glutamine and glutamate - their central role in cell metabolism and function. Cell Biochem Funct 2003;21:1–9. 10.1002/cbf.1003 12579515

[65] Shinzato C , Inoue M, Kusakabe M. A snapshot of a coral “holobiont”: a transcriptome assembly of the scleractinian coral, *Porites*, captures a wide variety of genes from both the host and symbiotic zooxanthellae. PLoS One 2014;9:e85182. 10.1371/journal.pone.0085182 24454815 PMC3893191

[66] Fontecave M , Atta M, Mulliez E. S-adenosylmethionine: nothing goes to waste. Trends Biochem Sci 2004;29:243–9. 10.1016/j.tibs.2004.03.007 15130560

[67] Nakamura J , Straub K, Wu J et al. The glutamine hydrolysis function of human GMP synthetase: identification of an essential active site cysteine. J Biol Chem 1995;270:23450–5. 10.1074/jbc.270.40.23450 7559506

[68] Wakefield TS , Kempf SC. Development of host- and symbiont-specific monoclonal antibodies and confirmation of the origin of the symbiosome membrane in a cnidarian-dinoflagellate symbiosis. Biol Bull 2001;200:127–43. 10.2307/1543306 11341574

[69] Rosset SL , Oakley CA, Ferrier-Pagès C et al. The molecular language of the cnidarian–dinoflagellate symbiosis. Trends Microbiol 2021;29:320–33. 10.1016/j.tim.2020.08.005 33041180

[70] Cabral M , Anjard C, Malhotra V et al. Unconventional secretion of AcbA in *Dictyostelium discoideum* through a vesicular intermediate. Eukaryot Cell 2010;9:1009–17. 10.1128/EC.00337-09 20472692 PMC2901666

[71] Manjithaya R , Anjard C, Loomis WF et al. Unconventional secretion of Pichia pastoris Acb1 is dependent on GRASP protein, peroxisomal functions, and autophagosome formation. J Cell Biol 2010;188:537–46. 10.1083/jcb.200911149 20156962 PMC2828923

[72] Qiao K , Wang M, Takano T et al. Overexpression of acyl-coa-binding protein 1 (ChACBP1) from saline-alkali-tolerant *Chlorella* sp. enhances stress tolerance in *Arabidopsis*. Front Plant Sci 2018;9:1772. 10.3389/fpls.2018.01772 30555504 PMC6282033

[73] Barott KL , Venn AA, Perez SO et al. Coral host cells acidify symbiotic algal microenvironment to promote photosynthesis. Proc Natl Acad Sci U S A 2015;112:607–12. 10.1073/pnas.1413483112 25548188 PMC4299235

[74] Robinson WD , Park J, Tran HT et al. The secreted purple acid phosphatase isozymes AtPAP12 and AtPAP26 play a pivotal role in extracellular phosphate-scavenging by *Arabidopsis thaliana*. J Exp Bot 2012;63:6531–42. 10.1093/jxb/ers309 23125358 PMC3504502

[75] Wang X , Balamurugan S, Liu SF et al. Hydrolysis of organophosphorus by diatom purple acid phosphatase and sequential regulation of cell metabolism. J Exp Bot 2021;72:2918–32. 10.1093/jxb/erab026 33491071

[76] Rosset S , Wiedenmann J, Reed AJ et al. Phosphate deficiency promotes coral bleaching and is reflected by the ultrastructure of symbiotic dinoflagellates. Mar Pollut Bull 2017;118:180–7. 10.1016/j.marpolbul.2017.02.044 28242282 PMC5441187

[77] Oakley CA , Newson GI, Peng L et al. The *Symbiodinium* proteome response to thermal and nutrient stresses. Plant Cell Physiol 2022;64:433–47. 10.1093/pcp/pcac175 PMC10109209 36565060

[78] Ezawa T , Hayatsu M, Saito M. A new hypothesis on the strategy for acquisition of phosphorus in arbuscular mycorrhiza: up-regulation of secreted acid phosphatase gene in the host plant. Mol Plant-Microbe Interact 2005;18:1046–53. 10.1094/MPMI-18-1046 16255243

[79] Rands ML , Loughman BC, Douglas AE. The symbiotic interface in an alga-invertebrate symbiosis. Proc R Soc B 1993;253:161–5. 10.1098/rspb.1993.0097

[80] Jackson AE , Miller DJ, Yellowlees D. Phosphorus metabolism in the coral-zooxanthellae symbiosis: characterization and possible roles of two acid phosphatases in the algal symbiont *Symbiodinium* sp. Proc R Soc B 1989;238:193–202.

[81] Almaguer C , Cheng W, Nolder C et al. Glycerophosphoinositol, a novel phosphate source whose transport is regulated by multiple factors in *Saccharomyces cerevisiae*. J Biol Chem 2004;279:31937–42. 10.1074/jbc.M403648200 15145930

[82] Ziegler P , Nikiforov A, Robinson B et al. Elevated uptake of glycerophosphoinositol through the Git1 permease causes cell growth inhibition in *Saccharomyces cerevisiae*. FASEB J 2017;31:781.16. 10.1096/fasebj.31.1_supplement.781.1627836987

[83] Slaby BM , Hackl T, Horn H et al. Metagenomic binning of a marine sponge microbiome reveals unity in defense but metabolic specialization. ISME J 2017;11:2465–78. 10.1038/ismej.2017.101 28696422 PMC5649159

[84] Kamke J , Sczyrba A, Ivanova N et al. Single-cell genomics reveals complex carbohydrate degradation patterns in poribacterial symbionts of marine sponges. ISME J 2013;7:2287–300. 10.1038/ismej.2013.111 23842652 PMC3834845

[85] Walker NS , Fernández R, Sneed JM et al. Differential gene expression during substrate probing in larvae of the Caribbean coral *Porites astreoides*. Mol Ecol 2019;28:4899–913. 10.1111/mec.15265 31596993 PMC6900098

[86] Jeong HJ , Du YY, Kang NS et al. Heterotrophic feeding as a newly identified survival strategy of the dinoflagellate *Symbiodinium*. Proc Natl Acad Sci U S A 2012;109:12604–9. 10.1073/pnas.1204302109 22814379 PMC3412036

[87] Chen MC , Hong MC, Sen HY et al. ApRab11, a cnidarian homologue of the recycling regulatory protein Rab11, is involved in the establishment and maintenance of the Aiptasia-*Symbiodinium* endosymbiosis. Biochem Biophys Res Commun 2005;338:1607–16. 10.1016/j.bbrc.2005.10.133 16288726

[88] Fransolet D , Roberty S, Plumier JC. Establishment of endosymbiosis: the case of cnidarians and *Symbiodinium*. J Exp Mar Biol Ecol 2012;420-421:1–7. 10.1016/j.jembe.2012.03.015

[89] Yoshioka Y , Yamashita H, Suzuki G et al. Whole-genome transcriptome analyses of native symbionts reveal host coral genomic novelties for establishing coral-algae symbioses. Genome Biol Evol 2021;13:1–18. 10.1093/gbe/evaa240 PMC7850063 33185681

[90] Yuyama I , Ishikawa M, Nozawa M et al. Transcriptomic changes with increasing algal symbiont reveal the detailed process underlying establishment of coral-algal symbiosis. Sci Rep 2018;8:16802. 10.1038/s41598-018-34575-5 30429501 PMC6235891

[91] Seto S , Tsujimura K, Koide Y. Rab GTPases regulating phagosome maturation are differentially recruited to mycobacterial phagosomes. Traffic 2011;12:407–20. 10.1111/j.1600-0854.2011.01165.x 21255211

[92] Perez S , Weis V. Cyclophilin and the regulation of symbiosis in *Aiptasia pallida*. Biol Bull 2008;215:63–72. 10.2307/25470684 18723638

[93] Ünal CM , Steinert M. Microbial peptidyl-prolyl cis/trans isomerases (PPIases): virulence factors and potential alternative drug targets. Microbiol Mol Biol Rev 2014;78:544–71. 10.1128/MMBR.00015-14 25184565 PMC4187684

[94] Klee CB , Ren H, Wang X. Regulation of the calmodulin-stimulated protein phosphatase, calcineurin. J Biol Chem 1998;273:13367–70. 10.1074/jbc.273.22.13367 9593662

[95] Dobson S , Bracchi V, Chakrabarti D et al. Characterization of a novel serine/threonine protein phosphatase (PfPPJ) from the malaria parasite, *Plasmodium falciparum*. Mol Biochem Parasitol 2001;115:29–39. 10.1016/S0166-6851(01)00260-2 11377737

[96] Shi X , Lin X, Li L et al. Transcriptomic and microRNAomic profiling reveals multi-faceted mechanisms to cope with phosphate stress in a dinoflagellate. ISME J 2017;11:2209–18. 10.1038/ismej.2017.81 28548660 PMC5607363

[97] Lin Z , Wang L, Chen M et al. The acute transcriptomic response of coral-algae interactions to pH fluctuation. Mar Genomics 2018;42:32–40. 10.1016/j.margen.2018.08.006 30197044

[98] Gierz SL , Forêt S, Leggat W. Transcriptomic analysis of thermally stressed *Symbiodinium* reveals differential expression of stress and metabolism genes. Front Plant Sci 2017;8:271. 10.3389/fpls.2017.00271 28293249 PMC5328969

[99] Keeling PJ , Eglit Y. Openly available illustrations as tools to describe eukaryotic microbial diversity. PLoS Biol 2023;21:e3002395. 10.1371/journal.pbio.3002395 37988341 PMC10662721

