# Supplementary figures and images for: Proteomes of native and non-native symbionts reveal responses underpinning host-symbiont specificity in the cnidarian–dinoflagellate symbiosis
Source: ISME J. 2024 Jul 11;18(1):wrae122. doi: 10.1093/ismejo/wrae122 (PMC11473927; doi:10.1093/ismejo/wrae122)

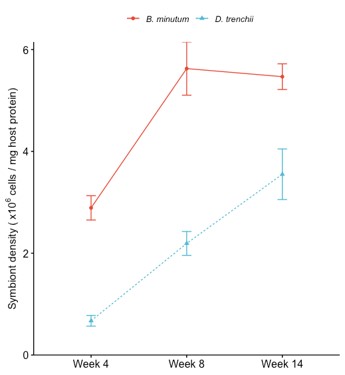

Supplement: Mashini_et_al_Supp_Figure_1_wrae122 [file mashini_et_al_supp_figure_1_wrae122.jpeg]
