# Supplementary material for: Proteomes of native and non-native symbionts reveal responses underpinning host-symbiont specificity in the cnidarian–dinoflagellate symbiosis
Source: ISME J. 2024 Jul 11;18(1):wrae122. doi: 10.1093/ismejo/wrae122 (PMC11473927; doi:10.1093/ismejo/wrae122)
Supplement: Mashini_et_al_Supp_Figure_1_Legend_wrae122 [file mashini_et_al_supp_figure_1_legend_wrae122.docx]

Figure S1. Population density of different symbiont species (*Breviolum minutum* and *Durusdinium trenchii*) in Aiptasia, at different time-points during 14 weeks of colonisation. ﻿Asterisks indicate significant differences between each time-point *vs.* Week 4: **p < 0.01, ***p < 0.001, ****p < 0.0001. Values are mean ± SE; n = 8 per symbiont species per time-point.
